# Exploring the Experiences, Perceptions and Social Dynamics of Electronic Cigarette Users: A Qualitative Study

**DOI:** 10.1111/hex.70066

**Published:** 2024-10-13

**Authors:** Ersan Gürsoy, Rıdvan Kaya

**Affiliations:** ^1^ Department of Family Medicine, Faculty of Medicine Erzincan Binali Yıldırım University Erzincan Turkey

**Keywords:** addiction, e‐cigarettes, electronic cigarettes, health perceptions, qualitative study, social dynamics, user experiences

## Abstract

**Introduction:**

Electronic cigarettes, or e‐cigarettes, are rapidly gaining popularity throughout the globe as safer alternatives to traditional cigarette smoking. There are significant public health concerns due to the uncertainty of long‐term health consequences. This study aims to examine the experiences, attitudes and social dynamics of e‐cigarette users to provide guidance for public health policies and interventions.

**Methods:**

In this qualitative descriptive study, semi‐structured interviews were conducted. Fifteen e‐cigarette users were recruited using a snowball sampling strategy, where initial participants referred other suitable users. All participants had at least 6 months of regular e‐cigarette use. Data were collected through face‐to‐face interviews with open‐ended questions. The interviews were transcribed verbatim and reviewed by the researchers. This review identified four main themes and eight sub‐themes.

**Results:**

The four main themes identified as a result of the analysis are as follows: (1) initiation and usage experiences; (2) perceptions of health effects; (3) social and environmental factors and (4) addiction and future plans. Participants primarily began using e‐cigarettes out of curiosity, due to the appealing fragrance and the belief that they were less harmful than traditional cigarettes. Usage patterns varied among participants, with some reporting minimal cravings in the early morning hours. Opinions on the long‐term health effects of e‐cigarettes were diverse, with many participants acknowledging uncertainty. Peers and family members perceived e‐cigarettes as more socially acceptable than traditional smoking, resulting in fewer negative reactions. Finally, participants' levels of addiction and intentions to quit varied, with some expressing a desire to reduce or cease usage due to health concerns.

**Conclusion:**

The findings of this study indicate that the adoption of e‐cigarettes is influenced by a confluence of factors, including curiosity, the perception of reduced damage in comparison to conventional cigarettes and social acceptance. Notwithstanding these claimed advantages, users have a diverse array of beliefs and understandings concerning the enduring health consequences of e‐cigarettes. The presence of varying levels of addiction and the corresponding aspirations to cease the behaviour highlight the necessity for focused public health interventions.

**Patient or Public Contribution:**

E‐cigarette users were actively involved in this study, providing essential insights and feedback throughout. Their first‐hand experiences shaped the interview guide and contributed to the identification of key themes. Participants also reviewed and confirmed the accuracy of the interview transcripts, ensuring the reliability of the data.

## 1 Introduction

The use of electronic cigarettes (e‐cigarettes), which have become a popular alternative to traditional smoking, is rapidly increasing worldwide [1]. Despite the promotion of e‐cigarettes as a safer alternative, the potential long‐term health consequences of their use remain unknown [2, 3]. The World Health Organization (WHO) has expressed serious concerns about the possible risks of e‐cigarettes, especially given their attraction to young people [4]. The WHO emphasises the need for strict regulation and increased public awareness to reduce these risks [4].

Despite these concerns, understanding the reasons behind the growing popularity of e‐cigarettes requires an in‐depth examination of users' experiences and perceptions [5]. In this context, the reasons for the initiation of consumption, patterns of consumption, levels of social acceptability and potential divergence from conventional tobacco products are of prime relevance in the formulation of meaningful public health policies [6, 7]. Moreover, knowledge of users' perceptions of the potential long‐term health consequences associated with the use of such products and the subsequent development of awareness campaigns for such consequences can serve as a trigger for efforts to stop the use of such products [8].

Current quantitative studies provide valuable epidemiological and behavioural insights into the prevalence and patterns of e‐cigarette use [1, 7, 9]. However, due to the nature of quantitative research, these studies are unable to address deeper questions related to the nuanced, personal experiences, motivations and perceptions of users. In this context, a qualitative approach may be more suitable [10]. In Turkey, where e‐cigarette importation and online sales are restricted, these legal constraints add a unique dimension to user behaviour and perceptions that has yet to be fully explored. Although the literature includes many studies exploring the experiences of e‐cigarette users, to the best of our knowledge, no such study has been conducted in Turkey, where e‐cigarette importation and online sales are legally restricted, and yet, their popularity continues to rise.

The primary objective of this study is to conduct a comprehensive analysis of the complexities of e‐cigarette users' experiences within this specific population, with the goal of elucidating the dynamics underlying the popularity of these products. By doing so, the study aims to contribute to the development of strategies designed to mitigate the potential public health threats posed by e‐cigarettes.

## 2 Materials and Methods

### 2.1 Research Design

This study utilised a qualitative descriptive design using semi‐structured interviews to explore the experiences and perceptions of e‐cigarette users. The aim was to capture first‐hand encounters and understand motivations, health perceptions and social interactions related to e‐cigarette use. The investigation was conducted in full conformity with the COREQ criteria, ensuring the credibility of the study findings [11].

### 2.2 Study Setting and Sample

The study was conducted at the Family Medicine Clinic of Erzincan Binali Yıldırım University Mengücek Gazi Training and Research Hospital. To ensure that all participants had sufficient exposure to vaping, individuals who had used e‐cigarettes regularly for at least 6 months were included in the study, and a snowball sampling approach was used [12]. Participants who visited the clinic for any reason and were identified as e‐cigarette users were asked to refer other users like themselves. This approach was chosen due to the difficulty in reaching users, as the import and online sale of these products are prohibited, making it challenging to access a broad sample. Although the study did not specifically focus on individuals who had experienced negative impacts from e‐cigarette use, it aimed to capture a wide range of experiences, both positive and negative. Vaping participants were interviewed in the senior author's office. By utilising the snowball sampling method, the study ensured a diverse and representative sample until data saturation was achieved, with a total of 15 individuals participating.

### 2.3 Data Collection Method

The main investigator gathered the data by conducting in‐person interviews with structured interview methodologies. The questions, derived from a comprehensive analysis of existing research, were posed to one participant at a time. Every interview, which lasted approximately 10–15 min, was recorded using audio technology with the participants' clear and direct permission. As each interview was conducted individually, the responses were free from external influences, thereby ensuring the authenticity and reliability of the data collected.

### 2.4 Procedure and Data Analysis

The interviews were transcribed verbatim, ensuring that every detail of the participants' responses was accurately captured. Initially, the two researchers independently reviewed the first few transcripts to identify potential themes. These themes were then compared and discussed in a meeting, where a consensus was reached on the definition of each theme. The initial codes, along with clear definitions and examples for coding, were incorporated into a codebook. To test the applicability of the codebook, a pilot study was conducted on the same transcripts. Any discrepancies in coding were resolved through discussion among the researchers, leading to the finalisation of the codebook. The two researchers independently coded all transcripts using the collaboratively developed codebook to identify the main themes and sub‐themes. The transcribed interviews were provided to participants for accuracy verification, but they were not involved in the coding process. At this stage, no participant disapproved of the transcribed texts. Quality checks were performed by comparing the codes assigned by each researcher, with any inconsistencies resolved through discussion until consensus was achieved. This iterative process continued until data saturation was reached, meaning no new themes emerged from additional data.

**Figure 1:**
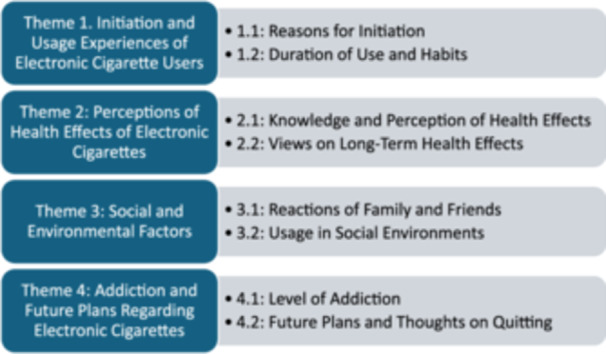
Themes and sub‐themes.

The final analysis identified four main themes and eight sub‐themes, reflecting the diverse experiences and perceptions of e‐cigarette users. These themes were categorised as follows: (1) initiation and usage experiences; (2) perceptions of health effects; (3) social and environmental factors (4) and addiction and future plans (Figure 1). NVivo software was utilised to facilitate the content analysis, enabling the systematic organisation and identification of these themes.

### 2.5 Reliability

The data's dependability was guaranteed by their credibility, reliability, confirmability, transferability and transparency. A semi‐structured interview guide was created using the literature and a conceptual framework. Various data‐gathering approaches were used, such as conducting individual interviews and taking observation notes. Volunteers served as informants and provided valuable insights into their experiences. The interviews were conducted individually in an environment where there were two people: a participant and the principal researcher (the principal researcher's office). The interviews were transcribed word for word, and participant verification (member checking) was utilised to enhance data reliability. Each participant was individually contacted, and their respective transcripts were sent to them. Participants were asked to review these transcripts to ensure that their statements were accurately represented. Feedback from participants was collected via SMS or email, and all 15 participants confirmed the accuracy of their transcripts.

### 2.6 Ethical Considerations

The study obtained ethical approval from the Clinical Research Ethics Committee of Erzincan Binali Yıldırım University (clearance No. 2023‐23/13), and all participants were obligated to submit written informed consent before the study's commencement. The transcripts were devoid of identification particulars, and the participants maintained anonymity while their confidentiality was preserved. Participants provided informed consent for the study methodology and materials and were advised of their right to withdraw their participation at any point. The updated Helsinki Declaration was followed during all phases of the investigation.

## 3 Results

This section presents themes and sub‐themes from the interviews. A summary of the main findings is presented in Table 1.

**Table 1:** Summary of key findings on electronic cigarette use.

Theme	Sub‐theme	Key findings^a^
Initiation and usage experiences	Reasons for initiation	Quitting traditional cigarettes (8), curiosity (6), aroma (5), influence from friends (5), no bad smell (4), lighter feeling (1), less harmful (1)
	Duration of use and habits	Daily use (15), no urge to smoke in the early morning (8), struggles with addiction (8)
Perceptions of health effects	Knowledge and perception	No significant harm felt (10), misconceptions about health impact (9), based on personal experience and hearsay (9), lack of sufficient knowledge (8)
	Views on long‐term effects	Uncertain or positive views (7), varying beliefs about long‐term effects (6), healthcare professional insights (2)
Social and environmental factors	Reactions of family and friends	Generally, positive or neutral reactions (11), normal acceptance in social circles (7), some curiosity from family and friends (2)
	Usage in social environments	More comfortable and acceptable social experiences (7), less bad odour (5), can be used in enclosed spaces (5), positive impact on social life (4)
Addiction and future plans	Level of addiction	Do not feel significant addiction (5), find it as addictive as traditional cigarettes (3), increased use compared to traditional cigarettes (3)
	Future plans and thoughts	Plan to quit or reduce usage (9), plan to quit if health concerns arise (5), do not plan to quit (5), mixed success with quitting (4)

^a^ The numbers in parentheses indicate the number of participants who mentioned the respective finding.

### 3.1 Theme 1: Initiation and Usage Experiences of E‐Cigarette Users

#### 3.1.1 Sub‐Theme 1.1: Reasons for Initiation

Most participants began using e‐cigarettes with the intention of quitting traditional cigarettes. For example, ‘Participant 1’ mentioned, ‘I started e‐cigarettes to quit classic cigarettes. I did not do research, obviously, but I started to quit smoking. I'm only vaping right now’. Similarly, ‘Participant 5’ expressed, ‘My reason for starting e‐cigarettes was to reduce or even completely quit normal cigarettes. I also started because the smell is better than normal cigarettes’. ‘Participant 13’ also noted, ‘The reason I started e‐cigarettes was to quit normal cigarettes completely. The difference from classic cigarettes is that both the aroma and smell are more beautiful’.

Some participants mentioned that they started using e‐cigarettes out of curiosity. ‘Participant 9’ stated, ‘I did not have any expectations or hopes. I just started out of curiosity. I did not do any research’. ‘Participant 8’ also stated that they started using e‐cigarettes out of curiosity. ‘I started out of curiosity. The reason I prefer e‐cigarettes is that they do not cause bad breath. I can vape in any environment. I started because of these advantages’.

#### 3.1.2 Sub‐Theme 1.2: Duration of Use and Habits

The duration of use and habits among e‐cigarette users are presented in Table 2.

**Table 2:** Estimated usage metrics and duration of smoking and e‐cigarette use.

Participant	Age	Years of smoking (regular)	Years of smoking (e‐cigarette)	Daily puff count^a^
1	23	2	0.5	~90
2	23	3	1	~120
3	24	7	2	~100
4	24	6	0.5	~80
5	26	7	2	~90
6	24	6	1	~100
7	25	7	1	~150
8	28	13	1	~80
9	29	7	5	~120
10	32	7	2	~60
11	31	13	2	~140
12	27	10	1	~100
13	29	8	3	~90
14	27	12	2	~80
15	25	8	2	~160

^a^ The approximate values are derived from an in‐depth inquiry into the participants' usage habits.

### 3.2 Theme 2: Perceptions of the Health Effects of E‐Cigarettes

#### 3.2.1 Sub‐Theme 2.1: Knowledge and Perception of Health Effects

Many participants indicated a lack of sufficient knowledge regarding the health effects of e‐cigarettes. ‘Participant 6’ stated, ‘In the long run, I do not think that e‐cigarettes will cause as much harm as normal cigarettes, I say this completely according to my estimation because it feels lighter’ (Participant 6 expressed uncertainty). ‘Participant 11’ mentioned, ‘It may cause shortness of breath or pulmonary edema in the future. I heard this from people around me who use it. So word of mouth information’. ‘Participant 12’ shared, ‘I think that the effects on health are greater than those of other normal cigarettes. I say this information from my own experience’.

#### 3.2.2 Sub‐Theme 2.2: Views on Long‐Term Health Effects

Participants generally have uncertain or positive views regarding long‐term health effects. ‘Participant 14’ stated, ‘In the long term, I think it will have less effect than normal cigarettes’. ‘Participant 8’ noted, ‘In the long term, I think that it may cause lung edema, as I look at the event as a health professional’ (Participant 8 appeared concerned from a professional standpoint). ‘Participant 7’ explained, ‘I have been using it for a year. I have not seen a big effect at the moment, but I believe that I will probably see much less effect than classic cigarettes. It made a difference even in a year’.

### 3.3 Theme 3: Social and Environmental Factors

#### 3.3.1 Sub‐Theme 3.1: Reactions of Families and Friends

E‐cigarette users generally receive positive or neutral reactions from their families and friends. ‘Participant 3’ mentioned (about relatives) ‘I think they are happier (with my use of e‐cigarettes) compared to normal cigarettes. I have not heard any bad comments about the smell and effects’ (Participant 3 seemed really happy in terms of social acceptance). ‘Participant 10’ stated, ‘I have not encountered any abnormal reactions thus far; I do not think that I am perceived neither positively nor negatively by society’. ‘Participant 9’ expressed, ‘I am perceived quite normally both by my family and friends. I think I am intrigued by society’.

#### 3.3.2 Sub‐Theme 3.2: Usage in Social Environments

E‐cigarette users often experience a more comfortable and acceptable experience in social settings. ‘Participant 7’ stated, ‘It was very advantageous in social life. I mean, most people were disturbed by the smell, but with this, people are not disturbed because there are flavors and so on. It can be closer. People do not move away. I can drink indoors; it has such advantages. I can say that it has been better in my social life’. ‘Participant 15’ noted, ‘I think it should not be used in social environments in order to set the right example. It does not restrict my social life as I can drink in closed environments’.

### 3.4 Theme 4: Addiction and Future Plans Regarding E‐Cigarettes

#### 3.4.1 Sub‐Theme 4.1: Level of Addiction

The level of addiction among e‐cigarette users varies. ‘Participant 3’ stated, ‘I think it is as addictive as a normal cigarette’. ‘Participant 12’ shared, ‘At first, I used it very little, that is, I used it rarely; after a certain period of time, I started to increase it. As I said before, I reduced it again because it caused shortness of breath and chest pain. I use it for an average of 1 hour or 2 hours a day at most’ (Participant 12 appeared uncomfortable with the increased use).

#### 3.4.2 Sub‐Theme 4.2: Future Plans and Thoughts on Quitting

Many participants had plans to quit or reduce their use of e‐cigarettes. ‘Participant 9’ mentioned, ‘I do not plan to quit for the time being, but I will quit when I think or feel that it is harmful to my health’. ‘Participant 12’ noted, ‘I am actually thinking of quitting e‐cigarettes completely rather than reducing them. Because when I think about the future and as a result of my research, of course, after using it, I think I will quit because I have observed that it is not the right thing; it is even more harmful than normal cigarettes’. ‘Participant 14’ explained, ‘Since my e‐cigarette use is decreasing daily, I plan to end the use of e‐cigarettes in the coming years, maybe after 6 months or a year’.

These findings comprehensively reveal the experiences, perceptions and future plans of e‐cigarette users, highlighting the need for further research on this topic. Participants' statements show that e‐cigarettes have different effects on users and that personal experiences are crucial for understanding these effects.

## 4 Discussion

The study's findings provide a comprehensive understanding of e‐cigarette use, showcasing the diverse elements that impact the start of consumption, patterns of use, perceptions of health and social interactions among users. The intricate and varied nature of user experiences highlights the necessity for sophisticated public health strategies and customised interventions.

### 4.1 User Experiences

The study emphasised that a significant number of individuals began using e‐cigarettes because they believed that it was a safer option than traditional smoking. Their goal was to either quit or decrease their use of regular cigarettes. This finding is consistent with other studies indicating that e‐cigarettes are frequently advertised and viewed as instruments for reducing harm [13, 14, 15]. However, it is important to note that this perceived safety is not fully supported by scientific evidence. Recent studies suggest that although e‐cigarettes expose users to fewer toxic substances compared to combustible cigarettes and have even been shown to improve certain pulmonary function test parameters compared to traditional smoking, they are not without harm [13, 16]. In particular, e‐cigarettes pose significant health risks, especially concerning respiratory and cardiovascular health [17, 18]. Moreover, the perception of reduced harm, combined with the ability to use e‐cigarettes more freely in social settings, may lead to frequent but smaller exposures to nicotine and other harmful substances. This pattern of use could potentially result in greater overall harm over time, as users may underestimate the cumulative risks associated with frequent vaping [2, 5, 8]. Nevertheless, in addition to health considerations, curiosity and social influences also have substantial impacts on initiation, suggesting that motives go beyond just health concerns.

### 4.2 Health Effect Perceptions

A significant finding of this study is the general lack of certainty and diverse opinions regarding the health impacts of e‐cigarettes. Although certain users perceive e‐cigarettes as less deleterious than conventional cigarettes, others are uncertain or perhaps regard them as more hazardous based on personal experiences and anecdotal data. Differences in perceptions about the safety and health effects of e‐cigarettes are in line with the conflicting views observed in the literature [19, 20]. The hypothesis that e‐cigarettes are a safer alternative to traditional cigarettes is generally based on the assumption of lower addiction potential and reduced risk profile of these products. However, current research points to a risk of developing severe nicotine dependence, especially in the younger population, due to the high nicotine concentrations in some e‐cigarette products [21, 22].

Recent studies suggest that e‐cigarettes have the potential to reduce exposure to certain harmful substances found in conventional tobacco smoke [23]. However, information on the long‐term health consequences of the vaporisation process is still limited and potential effects are thought to be significant [23]. In this context, more comprehensive and long‐term research is needed to fully understand the health effects of e‐cigarettes.

### 4.3 Socioenvironmental Determinants

The study revealed that the majority of society has a positive or neutral reaction to the use of e‐cigarettes. Users reported receiving more social acceptance while using e‐cigarettes than when using traditional cigarettes. This social acceptability is strongly influenced by the possibility to use e‐cigarettes in places where smoking is prohibited (e.g., indoors) without emitting bad odours [3]. Studies indicate that a significant number of users view e‐cigarettes as less harmful than traditional cigarettes, therefore enhancing their social acceptance [9, 24]. However, it is essential to recognise that these perceived social benefits may contribute to the normalisation of e‐cigarette use, particularly, among younger populations, which could undermine efforts to control nicotine addiction [19]. Studies indicate that the widespread acceptance of e‐cigarettes, especially in social settings, can lead to a false sense of security among users, potentially downplaying the risks associated with nicotine addiction and long‐term health effects [25]. In conclusion, although the participants' revelations about the social acceptability and perceived benefits of e‐cigarettes are valid, they may underestimate the potential risks and overestimate the benefits. The normalisation of e‐cigarette use, particularly among youth, remains a significant public health concern.

### 4.4 Substance Dependence and Long‐Term Goals

The participants' diverse degrees of addiction and their future intentions regarding the usage of e‐cigarettes suggest a multifaceted connection with these items. Several users reported experiencing a substantial level of addiction, similar to that of regular cigarettes, but others did not perceive a strong sense of addiction. This contrast implies that variations among individuals, such as their personal motives, patterns of usage and possibly genetic predispositions, have significant impacts on the levels of addiction.

In addition, the desire to quit or reduce e‐cigarette use was commonly expressed among participants. However, when we look at the participants, we see that all of them are both vaping and smoking. The ongoing inability to quit, leading to the concurrent use of both e‐cigarettes and traditional cigarettes, aligns with what is referred to in the literature as the ‘dual use pattern’ [26]. In this pattern, users become dependent on e‐cigarettes, whose long‐term health effects remain uncertain, while simultaneously continuing to expose themselves to the well‐documented harmful effects of traditional cigarettes [26]. For participants trapped in this cycle of dependence, there is a critical need for targeted public health interventions designed to support their cessation efforts [27].

### 4.5 Limitations and Potential for Future Research

This study has several limitations that may affect the generalisability and depth of the findings. The small sample size and reliance on participants from a single clinic restrict the applicability of the results to broader populations. The use of audio recordings rather than video may have limited the ability to capture the full emotional and non‐verbal context of participants' experiences. Additionally, the inclusion criteria for the study were quite narrow due to the difficulty of finding participants due to legal restrictions in Turkey and the reluctance of those participants to participate in the study for similar reasons, which could lead to ignoring a wider range of user experiences. Although participant verification was conducted through transcript reviews, the overall findings were not shared with participants for further input, which could have enhanced data triangulation. Future research should aim for more diverse samples, incorporate richer data collection methods and include longitudinal studies to better understand the long‐term health effects of e‐cigarettes and the dynamics of dual use.

## 5 Conclusion

Given the complex interplay of factors influencing e‐cigarette use, including perceptions of reduced harm, social acceptance and the reality of nicotine addiction, it is clear that a nuanced approach is required to address the public health implications of e‐cigarettes. Although some participants view e‐cigarettes as a safer alternative to traditional smoking, this perception is not fully supported by scientific evidence. Many users observe a dual‐use pattern, using e‐cigarettes alongside traditional cigarettes, which raises significant concerns about continued nicotine dependence and exposure to the harmful effects of both products.

Therefore, public health strategies must focus not only on educating the public about the potential risks of e‐cigarettes but also on implementing stricter marketing regulations to prevent the normalisation of e‐cigarette use, especially among younger populations. Interventions should aim to reduce dual use and support cessation efforts, acknowledging that e‐cigarettes are not a harmless alternative to smoking but rather a product that still poses significant health risks.

## Author Contributions

**Ersan Gürsoy:** conceptualisation, investigation, writing–original draft; methodology, writing–review and editing, software, formal analysis and Supervision. **Rıdvan Kaya:** conceptualisation; data curation, formal analysis, investigation, writing–original draft and software.

## Ethics Statement

The study obtained ethical approval from the Clinical Research Ethics Committee of Erzincan Binali Yıldırım University (No. 2023‐23/13).

## Consent

Informed consent was obtained from all individual participants included in the study.

## Conflicts of Interest

The authors declare no conflicts of interest.

## Data Availability

The data sets generated and analysed during the current study are not publicly available due to restrictions imposed by the local clinical ethics committee but are available from the corresponding author on reasonable request.
